# Cervical Abscesses in Children

**Published:** 1872-05

**Authors:** 


### CERVICAL ABSCESSES IN CHILDREN.

Mr. Richard Crean, L. K. Q. C. P. I., etc., writes to the *Medical Press and Circular*, October 5, 1871:

Two classes of cervical abscesses are met with in children. In one suppuration occurs in the tissues around the lymphatic glands, and its progress, though not rapid, is marked by an activity and vigor not characteristic of the other. In the second form tubercle is usually deposited in the substance of the gland, and a slow and painful enlargement follows. Sooner or later, in general, softening succeeds, and with tortoise-like pace, the abscess advances to the surface. The inflammatory symptoms are less intense. Pointing takes place, and instead of the homogeneous liquid usually found in the first variety, the discharged matter consists of cheesy flakes in a thin, turbid, and yellowish serum. Independently of the trouble, pain, and tediousness of the process, the physical difference in the contents of the abscesses would preclude the universal employment of tapping in these cases. Two cases lately under my care offer a marked illustration of this. In the first, tapping had the happiest effects; in the second, despite frequent and carefully made punctures with Wood's syringe, a solid, though small swelling remained after the evacuation of the fluid contents. A fortnight afterward inflammation of the gland recurred. It burst, and left a puckered scar. Short notes of a case published by Dr. Murray, of the Middlesex Hospital, in the *British Medical Journal*, last February, when he used a silk thread, and afterwards a piece of fine catgut steeped in carbolic acid, induced me to try a similar plan. In my first case I used silk, but found this acted as an irritant, extending the inflammatory process beyond its former limits. Next time I substituted silver wire and I found this answer its purpose so well that I have not cared to exchange it for catgut. I have now used it in twenty-eight cases, and in all it has afforded me great satisfaction. In a few weeks, sometimes in a few days, after the introduction of the wire, nothing remains to mark the site of the abscess except perhaps some induration, or slignt lividity of the skin, which in turn disappears.

Short notes of the two following cases will serve as an illustration of its advantages:

Case 1.—J. S., aged nine years, of marked strumous appearance, and suffering from chronic affections of the scalp and conjunctiva, was brought to the dyspensary on the 2d of April. An indurated gland, situated beneath the angle of the right jaw, after remaining quiescent for five months, had taken on suppurative action, and now formed an indolent abscess of fair size. A piece of fine silver wire was introduced, formed into a loop, and secured in position by means of a strip of adhesive plaster. In forty-eight hours the sac was completely emptied, and in a week all trace of it had disappeared.

Case 2.—A. M., aged three and a half years, convalescing from measles, is suffering from a painful induration in right submaxillary space. Quinine and poultices of fresh hemlock leaves were ordered, the latter of which I found a very efficient agent in dissipating painful glandular enlargements. In a week the centre of the tumor had softened, and was threatening to burst, while the circumference preserved a firm consistence. A wire was passed through the base of the abscess, and was left there for a week. On removing, a little thickening and hardness was still perceptible to the touch; but gentle friction for a short time with camphorated oil destroyed this.

